# Structure vs. Function of TRIB1—Myeloid Neoplasms and Beyond

**DOI:** 10.3390/cancers13123060

**Published:** 2021-06-19

**Authors:** Hamish D McMillan, Karen Keeshan, Anita K Dunbier, Peter D Mace

**Affiliations:** 1 Biochemistry Department, School of Biomedical Sciences, University of Otago, P.O. Box 56, Dunedin 9054, New Zealand; mcmha419@student.otago.ac.nz (H.D.M.); anita.dunbier@otago.ac.nz (A.K.D.); 2 Paul O’Gorman Leukaemia Research Centre, Institute of Cancer Sciences, University of Glasgow, Scotland G12 0YN, UK; Karen.Keeshan@glasgow.ac.uk

**Keywords:** Tribbles, TRIB1, TRIB2, leukaemia, breast cancer, prostate cancer, hepatocarcinoma

## Abstract

**Simple Summary:**

Tribbles proteins possess the structure of protein kinases but function by forming protein complexes rather than phosphorylating substrates. Here we review the structure–function relationship of TRIB1 in cancers. Of the Tribbles proteins, TRIB1 is currently the most well characterised structurally. TRIB1 has different states that could potentially be targeted by small-molecule inhibitors and well-established relevance in acute myeloid leukaemia through degradation of transcription factors. Less is understood about the role of TRIB1 in solid tumours. Further research is required to fully realise the potential of TRIB1 as either a direct target of small-molecule drugs or a biomarker of treatment response across diverse cancer types.

**Abstract:**

The Tribbles family of proteins—comprising TRIB1, TRIB2, TRIB3 and more distantly related STK40—play important, but distinct, roles in differentiation, development and oncogenesis. Of the four Tribbles proteins, TRIB1 has been most well characterised structurally and plays roles in diverse cancer types. The most well-understood role of TRIB1 is in acute myeloid leukaemia, where it can regulate C/EBP transcription factors and kinase pathways. Structure–function studies have uncovered conformational switching of TRIB1 from an inactive to an active state when it binds to C/EBPα. This conformational switching is centred on the active site of TRIB1, which appears to be accessible to small-molecule inhibitors in spite of its inability to bind ATP. Beyond myeloid neoplasms, TRIB1 plays diverse roles in signalling pathways with well-established roles in tumour progression. Thus, TRIB1 can affect both development and chemoresistance in leukaemia; glioma; and breast, lung and prostate cancers. The pervasive roles of TRIB1 and other Tribbles proteins across breast, prostate, lung and other cancer types, combined with small-molecule susceptibility shown by mechanistic studies, suggests an exciting potential for Tribbles as direct targets of small molecules or biomarkers to predict treatment response.

## 1. Introduction

Pseudokinases, proteins that adopt a protein kinase fold but are incapable of catalysing phosphorylation, are estimated to comprise ~10% of the human protein kinome [1,2,3]. Far from being dead remnants, pseudokinases specialise in a variety of other roles, including allosteric regulation of catalytically active partners, scaffolding protein–protein interactions or acting as signalling switches [1,4]. Because of their ability to regulate diverse signalling pathways, many pseudokinases are relevant to cancer progression, therapeutic response and the development of novel future therapies [5]. 

The Tribbles proteins are a family of pseudokinases that regulate diverse aspects of cellular signalling and metabolism [6]. The family in humans comprises three core members—TRIB1, TRIB2 and TRIB3—and the related STK40 protein. STK40 is somewhat more divergent in terms of sequence but has overlapping structural and functional features (described below). Tribbles are strongly implicated in the normal differentiation of myeloid cells [7,8] and adipocytes [9]. Correspondingly, their dysregulation can impact myeloid neoplasms and metabolic disruption in cancer. However, there is emerging evidence that Tribbles also contribute to development and therapeutic resistance in solid tumours. 

The structure and function of Tribbles proteins are intimately linked to whether the proteins can be targeted for therapy, or as biomarkers, across different forms of cancer. However, it is not clear whether the mechanisms by which Tribbles contribute to myeloid neoplasms are the same as or distinct from those by which Tribbles function in other cancer types. Here we describe our current knowledge of how TRIB1 structure and function are linked in the context of cancer. While all Tribbles proteins have cancer-specific roles, we focus on TRIB1 for two reasons. Firstly, TRIB1 underpins a large proportion of our current understanding of Tribbles structure, and thus our ability to identify potential therapies. Secondly, the gene encoding TRIB1 shares a close genetic linkage with the *c-MYC* oncogene, which could make it relevant to solid tumours where Tribbles function is currently less well understood. 

## 2. Molecular Mechanism of Tribbles Function

### 2.1. Overall Tribbles Structure 

**Figure 1:**
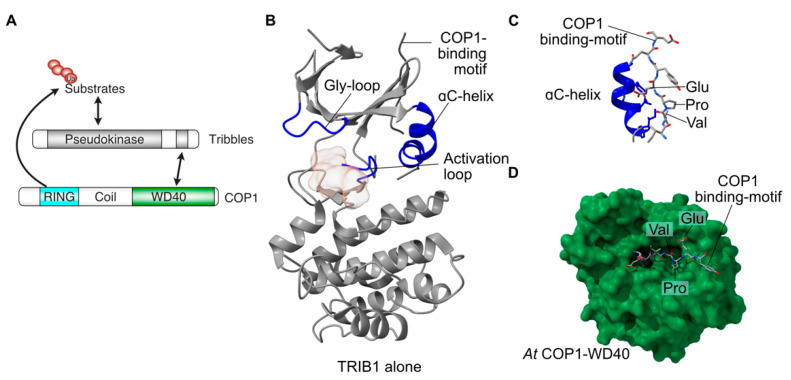
Structure and function of TRIB1. (**A**) Schematic of the predominant role of Tribbles proteins, bringing together substrates and COP1 to promote substrate ubiquitination. (**B**) Structure of autoinhibited TRIB1 (grey; PDB 5cem). Key features are coloured blue and labelled. (**C**) Close-up view of the C-terminal COP1-binding motif binding to the back of the ɑC-helix in autoinhibited TRIB1. (**D**) Structure of the C-terminal COP1-binding motif bound to the WD40 domain of COP1 (PDB 5igq).

The three mammalian Tribbles homologs and STK40 share a common domain architecture, with a central kinase-like domain flanked by N- and C-terminal extensions (Figure 1A). The N-terminal extensions are least well conserved amongst the family and have been proposed to determine protein localisation [10,11]. The central kinase-like domains share a large degree of sequence identity, particularly amongst TRIB1–3, and are indispensable for Tribbles function [6,12,13], especially substrate recruitment. Within the C-terminal tail of TRIB1–3 and STK40 is a conserved motif that recruits the ubiquitin E3-ligase constitutive photomorphogenesis protein 1 (COP1) (Figure 1A) [6,12,14,15]. This motif is an important feature of the proposed mode of action of Tribbles proteins; by binding to the COP1 ubiquitin-ligase, and to substrates through their kinase-like domains, Tribbles proteins act as adapter molecules for ubiquitination of substrates by COP1. In general, Tribbles substrates include MAP kinases from both the ERK and JNK pathways [16,17,18,19], AKT [20,21,22,23,24], acetyl-CoA carboxylase [25] and CCAAT-enhancer-binding proteins (C/EBPs) [26,27,28].

### 2.2. Tribbles Pseudokinase Domain Structure

Pseudokinases by definition lack one or more features that are essential for the catalysis of phosphoryl transfer. In the case of Tribbles proteins, two key features normally required for ATP binding and catalysis differ in sequence from the canonical residues surrounding the active site (Figure 1). These features are a glycine-rich loop that normally caps the ATP binding site and a divergent sequence within the so-called ‘Asp-Phe-Gly’ motif, within which the aspartate residue coordinates magnesium to facilitate ATP binding and catalysis in conventional kinases. In TRIB1 the ‘Asp-Phe-Gly’ motif is replaced by Ser-Leu-Glu residues, with some minor variation in the sequence between Tribbles proteins (Figure 1A). Consistent with the loss of the aspartate, TRIB1 is not able to bind ATP. There are data showing that TRIB2 and TRIB3 retain some ability to bind ATP [29], but STK40 also appears to be devoid of ATP binding of nucleotides or divalent cations, both of which are essential for enzymatic activity [15]. With no, or low, affinity to bind ATP, the capacity of Tribbles proteins to carry out catalysis is clearly impaired. 

Two crystal structures of the TRIB1 pseudokinase domain have shown features that depart from those of conventional active kinases. The aforementioned Gly-rich loop that normally caps the active site is retracted in TRIB1, rather than an extended conformation seen in conventional kinases (Figure 1). Crucially, a key feature of the catalytic pocket of conventional kinases—the ɑC-helix—is malformed in TRIB1, incorporating a bent shape relative to the extended ɑ-helix (Figure 1B). An unexpected observation from the structure of a longer TRIB1 construct containing both the pseudokinase and the C-terminal COP1-binding motif was that the COP1-binding motif binds to the back of the deformed ɑC-helix [12]. Parallel structural studies have shown how the C-terminal tail of TRIB1 binds to COP1, with the Val-Pro-Glu motif from TRIB1 binding to a substrate-binding pocket of the COP1 WD40 domain (Figure 1D) [30]. Crucially, this binding mode is not compatible with the C-terminus of TRIB1 simultaneously binding to the ɑC-helix of the pseudokinase domain and the WD40 domain of COP1. In the context of TRIB1 pseudokinase function, this means that the TRIB1 pseudokinase domain can restrict the availability of the COP1-binding motif, creating an autoinhibited conformation. 

### 2.3. Co-ordination of Substrate- and COP1-Binding

**Figure 2:**
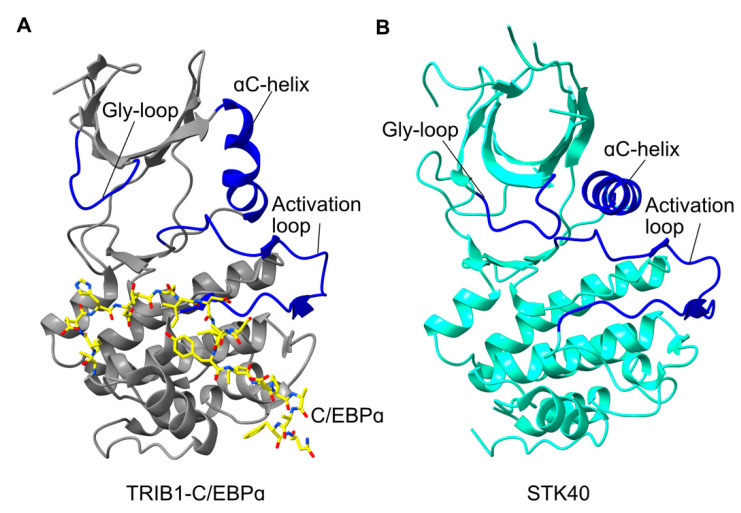
Comparison of activated TRIB1 structure with STK40. (**A**) Structure of TRIB1 (grey) in complex with a C/EBPɑ peptide (yellow; PDB 6dc0). (**B**) Structure of the STK40 pseudokinase domain (cyan; PDB 5l2q). Key features are shown in blue and labelled accordingly.

Rather than exhibiting catalytic activity, the pseudokinase domain of Tribbles family members binds to a range of different partner proteins. At this stage, our understanding of specific interactions is limited to that of a motif within C/EBPɑ [12]. The structure of the C/EBPɑ degron bound to TRIB1 shows that significant rearrangement is required from the autoinhibited structure observed for TRIB1 alone [14]. Most noticeably, the activation loop changes from a disordered conformation to an ordered conformation to generate the binding site for C/EBPɑ (Figure 2A). Stabilisation of the activation loop is tightly linked with a conformational change in the TRIB-specific Ser-Leu-Glu sequence, which allosterically promotes the release of the C-terminal COP1-binding motif. Namely movement of Leu226 within the Ser-Leu-Glu motif is accommodated by the movement of Tyr134 from the ɑC-helix, causing a conformational change in the ɑC-helix and disrupting residues that bind the C-terminal COP1-binding motif. Thus, substrate binding is allosterically linked with the release of TRIB1 autoinhibition—a mechanism that can link substrate- and COP1-binding. 

Of particular clinical significance is the movement of the ‘Ser-Leu-Glu’ sequence in the activation loop which opens the TRIB1 active site, making it a potential drug target [14]. The opening of this potential binding pocket is consistent with reports of TRIB2 being able to bind small-molecule ligands, as these proteins are closely related and likely share some structural features [14,31]. Initial screening of a kinase inhibitor library returned several promising compounds that stabilised TRIB1. Further compound optimisation is required to elaborate on leads for both TRIB1 and TRIB2, but knowing that TRIB1 can adopt SLE-in and SLE-out conformations analogous to DFG-in and DFG-out conformations of conventional kinases is highly relevant to future efforts to pharmacologically target Tribbles proteins. Compounds could potentially be designed to promote the release of the C-terminal tail, to recruit COP1 in the absence of substrate, to potentially promote ubiquitination and degradation. The ability to promote TRIB1 binding to COP1 without substrate, to promote its degradation, could have a significant impact because many of the disease states described in this review are related to TRIB1 overexpression. 

### 2.4. Structural Conservation of Tribbles Proteins

On the whole, sequence conservation amongst Tribbles proteins is concentrated within the pseudokinase domain. However, there do appear to be crucial differences that may affect the structure and function of other Tribbles proteins relative to TRIB1, hence representing features that could be exploited for potential therapeutics. For instance, the Tyr134 residue that is crucial to conformational switching of TRIB1 is not conserved in its closest relative TRIB2. Mutating Tyr134 in TRIB1 to its TRIB2 equivalent (cysteine) can destabilise TRIB1 [14]. This highlights that this region of the protein is crucial for conformational dynamics and potentially crucial for drug development. At the time of preparation, the only other Tribbles homolog protein to be structurally characterised is STK40 [15]. TRIB1 and STK40 are remarkably conserved within their C-terminal lobes but differ considerably in the N-terminal lobe. Most notably the ɑC-helix is relatively conventional in STK40, with an extended helix rather than the bent conformation seen in TRIB1. This suggests that STK40 may not recruit its own C-terminal tail, even though it contains a functional COP1-binding motif. Moreover, even though the STK40 structure was solved without substrate, the activation loop is in a conformation very similar to the C/EBPɑ-bound state of TRIB1. Overall, comparing TRIB1 with the structure of STK40 and other Tribbles proteins suggests that regulatory mechanisms, and potentially therapeutic susceptibility, could well be different between the Tribbles homologs, consistent with their varied biological roles. 

## 3. Cancer-Relevant Pathways Regulated by TRIB1 

### 3.1. Proposed Interaction Partners of Tribbles and TRIB1

Tribbles proteins have been proposed to bind to a wider range of proteins, including various kinases, transcription factors, ubiquitin ligases and other functional enzymes [6,32]. However, as the focus of this review is TRIB1 in cancer, the discussion of interaction partners is centred on purported TRIB1 interaction in cancer-associated signalling pathways. TRIB1 is purported to interact with proteins involved in a range of cancer-associated pathways, including cell cycle progression [21], invasion and migration [33], differentiation [7,34,35,36,37] and p53 activation [38,39]. TRIB1 interactors typically fall into one of two categories: substrate proteins, which include C/EBPs [12,26,27], p53 [39], MXLIPL [40], RARs [41] and SAP18 [42], or effector proteins, such as MEK1 [43], COP1 [12,26] and HDAC1 [39,44,45]. Substrate proteins typically have their function or stability directly altered by TRIB1 binding or are post-translationally modified by a TRIB1-recruited effector protein. This modification is commonly ubiquitination and substrate degradation, though more evidence for functional modulation is emerging. 

### 3.2. TRIB1 and MAP Kinase Pathway Regulation

The Tribbles family of proteins has been associated with a wide variety of kinase signalling pathways across cell types and species [6,20,21,43]. One group of kinase pathways purported regulated by TRIB1 are the MAP kinase (MAPK) signalling cascades, which are responsible for the integration of a wide variety of cellular stimuli. The MAPK signalling cascades target a number of different effector proteins, including ERK1/2, JNK and p38, which regulate a range of processes such as cell proliferation, differentiation and stress response. 

In acute myeloid leukaemia (AML), TRIB1 overexpression is associated with increased MEK1/ERK activity, which aids in promoting the characteristic enhancement of cell self-renewal, required for AML development [43]. TRIB1 is thought to regulate MEK1/ERK activity through a direct interaction with MEK1. This interaction occurs via a putative MEK1-binding motif at the beginning of the TRIB1 C-terminal tail [43]. Loss of the MEK1 motif significantly reduces the capacity of transplanted cells, overexpressing TRIB1, to induce AML in mice and substantially reduces ERK1/2 phosphorylation [43]. Further investigation of this purported interaction is required, as similar results may be expected if mutation or loss of the MEK1-binding motif inhibits COP1 recruitment. It has also recently been shown that the degradation of C/EBPα is the primary mechanism through which TRIB1 drives AML progression, with a more minor contribution from MEK1/ERK activation [46]. 

In AML, MAPK cascade activation of p38 is an essential component of the stress response, and its silencing or blunting is required to avoid cell cycle checkpoints and apoptosis. TRIB2 is an important regulator of p38 activation in AML stress response, with TRIB2 deficiency resulting in impaired p38 activation [16]. This contrasts with the oncogenic role of TRIB1 in MEK1/ERK signalling, suggesting TRIB2 acts as a tumour suppressor by promoting the activation of stress-activated, rather than proliferative, MAPK pathways in response to stress. These contradicting roles in stress response are supported by the inverse correlation in TRIB1 and TRIB2 expression in normal and malignant haematopoiesis [7]. TRIB2 expression is also associated with normal and stress-induced MAPK activation in thymocyte development and T-cell acute lymphoblastic leukaemia [47]. 

### 3.3. TRIB1 and AKT in NF-κB Regulation

AKT phosphorylation is critical in the regulation of a number of cellular processes, including cell cycle progression, metabolism and cell survival. One mechanism through which AKT regulates these processes is the regulation of NF-κB signalling. In *Drosophila*, Tribbles (Trbl) regulates AKT activity by directly binding and blocking activating phosphorylation [20,22]. Likewise, TRIB3 appears to have an inhibitory effect on AKT in the liver [24]. In contrast, TRIB1 appears to promote AKT1 activity, in line with the ability of TRIB2 [48]. TRIB1 knockdown in triple-negative breast cancer cells results in inhibited AKT1 phosphorylation and activity [21]. The inhibition of AKT1 leads to inhibited substrate phosphorylation that influences several cellular processes, including the activity of NF-κB [21]. Regulation of NF-κB, via AKT1 inhibition, is purported to be critical in TRIB1 regulation of the cell cycle and TRAIL drug response in triple-negative breast cancer cells [21]. NF-κB is associated with a wide variety of processes across cell types, including apoptosis, cell proliferation, inflammatory response and immune cell differentiation [49]. TRIB1 regulation of AKT1 and NF-κB seems likely to have a substantial impact on a variety of cellular processes across a range of cell and cancer types, though further investigation is required to understand the mechanism. 

### 3.4. TRIB1 in JAK/STAT Signalling 

The JAK/STAT signalling pathway is a potent kinase cascade responsible for transfer and amplification of external signals and therefore is critical in cellular response to external stimuli. One important role of JAK/STAT signalling is in immune cell differentiation. The polarisation of M1-like and M2-like macrophages is critical in innate immunity and tissue homeostasis and is partially regulated by JAK/STAT signalling [37,50]. In TRIB1-knockout bone-marrow-derived macrophages, JAK1 levels are lowered, leading to decreased phosphorylation and activation of STAT1, STAT3 and STAT6 which directly affects M1/M2 macrophage polarisation [51]. Additional regulation of C/EBPα or C/EBPβ by TRIB1 further compounds the effects of TRIB1 on macrophage polarisation and function. Such regulation has clear implications in the modulation of the tumour microenvironment, but JAK/STAT regulation may also have implications for tumour development and progression if it is conserved in other tissue types. The mechanism linking TRIB1 to JAK/STAT activity in tumour cells has yet to be identified, though further investigation is warranted.

### 3.5. TRIB1 in Retinoic Acid Signalling

Nuclear retinoic acid receptors (RARs) and retinoid X receptors (RXRs) are nuclear receptors that promote transcription in response to retinoid ligands [52]. Retinoic acid signalling is important in haemopoietic differentiation and is dysregulated in a number of haemopoietic cancers, including AML, acute promyelocytic leukaemia (APL) and T-cell lymphoma [52]. Retinoic acid signalling has also been implicated in a number of solid tumour types, including breast cancer, though its role is controversial [52]. TRIB1 has been implicated as a negative regulator of retinoic acid receptor activity [41]. The two types of nuclear receptors associated with retinoid signal transduction, RARs and RXRs, form a heterodimer in the presence of a ligand, such as all-trans retinoic acid (ATRA). An activated retinoic acid receptor directly binds DNA and recruits coactivators to promote transcription [41]. TRIB1 is able to bind to both receptors, in the presence or absence of a ligand, and is purported to negatively regulate the activity of the heterodimerised receptor by inhibiting the recruitment of coactivators, though the mechanism is not fully understood [41]. TRIB1 overexpression desensitises PML/RARA fusion-protein driven APL to ATRA treatment, suggesting that TRIB1 negatively regulates RAR/RXR activity [53]. While the regulation of retinoic acid signalling has implications across cancer types, the putative role of TRIB1 in ATRA resistance is particularly relevant in AML and APL, where ATRA is commonly used to drive cell differentiation as part of treatment.

## 4. TRIB1 Function in Cancer Development and Therapy

### 4.1. TRIB1 in Myeloid Neoplasms

Since the association of Trbl with cell proliferation in *Drosophila* development, the human Tribbles homologs have been implicated in a variety of human pathologies, including multiple cancers [6,21,27]. A major role of TRIB1 and TRIB2 is to regulate levels of C/EBP family transcription factors [27,28], which are major upstream regulators of proliferation and differentiation of haematopoietic cells [54,55,56,57]. Both TRIB1 and TRIB2 have been implicated as oncogenes in AML, with the independent overexpression of both genes found sufficient to drive leukaemogenesis in mice [27,28,43,46]. Regulation of C/EBPs appears to be the fundamental mechanism behind this oncogenic capability. The interactions behind the C/EBP regulation are the most extensively studied TRIB1 interactions and underpin TRIB1 function in a variety of contexts.

**Figure 3:**
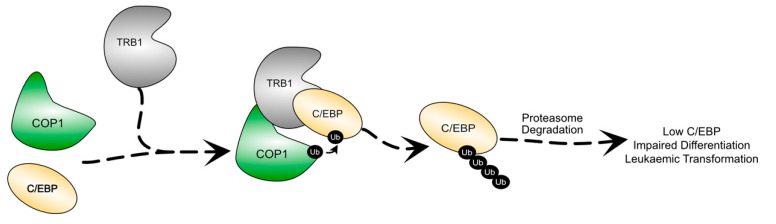
Schematic illustration of the degradation of C/EBP proteins by TRIB1. TRIB1 scaffolds the interaction between COP1 and C/EBP, resulting in the ubiquitination and proteasomal degradation of C/EBP. Lowered C/EBP levels are associated with impaired haematopoietic differentiation and leukaemic transformation.

In haematopoietic differentiation, TRIB1 mediates the degradation of C/EBPα (Figure 3) to control myeloid differentiation [7,8,36]. To facilitate the degradation of C/EBPα, TRIB1 scaffolds an interaction between C/EBPα and the E3 ubiquitin ligase, COP1 (Figure 3), leading to the ubiquitination of C/EBPα [12,14,26]. This mechanism of C/EBPα degradation is evolutionarily conserved from Drosophila where Trbl regulates the C/EBPα homolog Slbo through a similar ubiquitin-dependent mechanism [12,14,27,58]. The structural investigation of COP1–TRIB1–C/EBPα interaction was discussed earlier in this review. While both TRIB1 and TRIB2 have been shown to mediate C/EBPα degradation, suggesting redundancy, the strong inverse relationship between TRIB1 and TRIB2 expression in haematopoiesis suggests independent functions, though further investigation is required [7].

TRIB1 overexpression is important in homeobox a9 (Hoxa9)/murine ecotropic virus integration site 1 (Meis1)-mediated AML [46,59]. In Hoxa9/Meis1-driven AML, Hoxa9 is able to bind DNA as a transcription factor and in complex with Meis1 drives unregulated gene expression promoting leukaemogenesis [8,46,59]. The cellular defence against Hoxa9/Meis1-driven reprogramming is the colocalisation of C/EBPα and Hoxa9, which prevents the formation of the Hoxa9/Meis1 complex, suppressing the Hoxa9/Meis1 transcriptional programme. The overexpression of TRIB1 leads to enhanced C/EBPα degradation, removing this suppression, allowing Meis1 to interact with Hoxa9, promoting unregulated transcription [8,46,59]. Co-operation between Hoxa9/Meis1 and TRIB1 overexpression drives a more aggressive AML than TRIB1 overexpression alone [59]. The degradation of C/EBPα not only allows the formation of the Hox9/Meis1 transcriptional complex, but also results in the modification of Hoxa9-associated super-enhancers, which further enhances the transformative transcriptional programme [46]. 

As discussed above, TRIB1 overexpression results in enhanced MAPK pathway activation, which can impact AML development [19,43]. Two pieces of evidence suggest that MAPK regulation and C/EBPα-related effects in AML may be related: deletion of the MEK1-binding region of TRIB1 inhibits C/EBPα degradation [10,17,37], and C/EBPα degradation is inhibited by the MEK1 inhibitor U0126 [43,60]. The role of phosphorylation in C/EBPα degradation is not yet fully understood, but C/EBPβ does contain a MAPK target site that does not bind TRIB1 in its phosphorylated form [14]. While only tested *in vitro*, this may provide at least one mechanism by which MAPK pathways could regulate TRIB1-mediated C/EBPα/β degradation in AML. 

### 4.2. TRIB1 in Solid Tumours

While the importance of TRIB1 in myeloid neoplasms is well established, the function of TRIB1 in solid tumours is less well understood. However, TRIB1 has been associated with the development and progression of a number of solid tumour types, including breast cancer [21,39], hepatocellular carcinoma [38], glioma [45], gastric cancer [61], prostate cancer [62,63] and colorectal cancer [33]. The purported mechanisms of TRIB1 action vary across cancer types, with TRIB1 implicated in cell cycle regulation [21], p53 regulation [38,39,45] and microenvironment regulation [62], among other processes as discussed below. The apparent role of TRIB1 in a range of important cellular processes, across cancer types, highlights the need for further investigation of TRIB1 in solid tumours. The need for further investigation is further emphasised by the impact of TRIB1 amplification and overexpression on patient outcome in breast and colorectal cancers. In breast cancer, TRIB1 amplification is significantly associated with decreased breast cancer-specific and overall survival [21]. In colorectal cancer, TRIB1 overexpression is significantly associated with decreased disease-free survival [33]. 

#### 4.2.1. Regulation of p53

**Figure 4:**
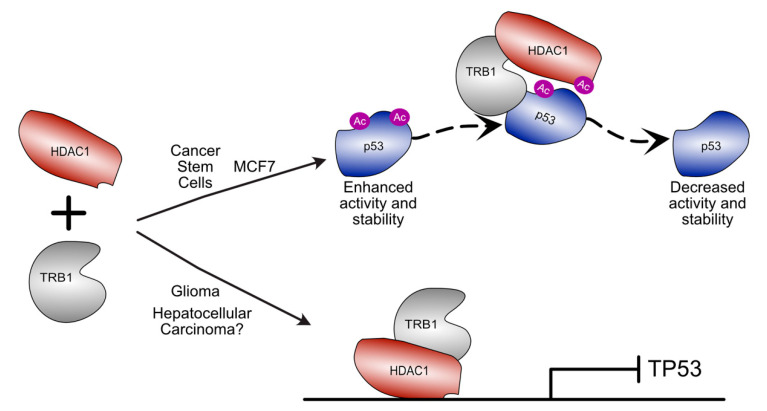
Schematic illustration of divergent roles for TRIB1–HDAC1 complexes in different cancer types. In cancer stem cells derived from non-small-cell lung cancer and in MCF7 breast cancer cells, TRIB1 can mediate destabilisation of p53 by scaffolding an interaction between HDAC1 and p53 resulting in p53 deacetylation. In glioma and hepatocellular carcinoma, TRIB1–HDAC1 has been shown to regulate TP53 expression. In glioma TRIB1 and HDAC1 colocalise to the TP53 promoter to modulate expression.

TRIB1 is associated with the regulation of p53 at the transcriptional and protein levels across cancer types [38,39,45]. In p53-stimulated MCF7 breast cancer cells, TRIB1 modulates p53 protein activity [39]. Knockdown of TRIB1 sensitises the cells to the Mdm2 antagonist nutlin-3, resulting in increased p53 activity [39]. In terms of mechanism, TRIB1 was found to mediate the formation of a complex with p53 and HDAC1, which purportedly results in the deacetylation of p53 [39] (Figure 4). Deacetylation of p53 destabilises the protein, decreasing its capacity for sequence-specific DNA binding [39]. This allows TRIB1 to prevent p53 activity, which is a hallmark of cancer progression [64]. The TRIB1-dependent deacetylation of p53 is an appealing model, as the function of TRIB1 as a scaffold is well established [12,14,26]. 

The downregulation of p53 activity, via TRIB1-mediated HDAC1-dependent deacetylation, is also thought to be key in the development of cancer stem cells after cisplatin treatment in non-small-cell lung cancer [44]. Cisplatin treatment upregulates C/EBPβ, leading to increased TRIB1 expression, resulting in enhanced deacetylation of p53. The inactivation of p53 by deacetylation promotes the formation of drug-resistant cancer stem cells, which are able to grow through multiple different drug treatments [44].

In contrast to the TRIB1–p53 relationship observed in MCF7 cells, in hepatocellular carcinoma cells, the expression levels of TRIB1 and TP53 are negatively correlated [38], suggesting an alternative mechanism of regulation. However, the acetylation state of p53 in hepatocellular carcinoma has not been examined [38]. In hepatocellular carcinoma cell lines, the knockdown of TRIB1 results in significantly enhanced TP53 expression [38]. The sensitivity of TP53 expression and protein levels to changes in TRIB1 expression suggests a transcriptional mechanism of TP53 regulation in hepatocellular carcinoma. While no specific mechanism for this transcriptional regulation is proposed, a feedback loop for the regulation of TRIB1 by miR23a was presented [38]. The in vivo knockdown of TRIB1 in hepatocellular carcinoma tumours in mice results in increased p53 levels, with significantly reduced tumour volume and increased cellular apoptosis relative to untreated tumours [38]. TRIB1 knockdown also downregulated β-catenin and c-MYC, which, combined with evidence of p53-dependent β-catenin stimulation by TRIB1, suggests TRIB1 promotes hepatocellular carcinoma tumour growth by removing p53-mediated tumour suppression [38].

A similar mechanism may be at play in glioma, where TRIB1 overexpression promotes radioresistance by repressing the expression of TP53 [45]. TP53 expression is regulated by a TRIB1–HDAC1 complex that binds the TP53 promoter region and represses expression (Figure 4) [45]. This may provide a mechanism through which TP53 expression could be regulated in hepatocellular carcinoma, though further investigation is required. 

#### 4.2.2. Association with the Oncogenic c-MYC

A broad-reaching mechanism for TRIB1 overexpression could be the location of TRIB1 within the common cancer amplicon 8q24, which also contains the oncogene *c-MYC* [17,53,60,65,66]. The selection for the co-amplification of TRIB1 and c-MYC, across cancer types, suggests dependency between the two genes. TRIB1 has been identified as a possible synthetic lethal partner of c-MYC overexpression [67,68], conceptually supporting selection for TRIB1/c-MYC co-amplification [69,70]. The c-Myc protein is a general transcriptional amplifier that functions across many cancer types and is strongly associated with aggressive tumourigenesis and poor clinical outcome [69,70,71]. The nature of c-Myc, as a general transcriptional amplifier, allows it to drive transformation in a wide range of cancers with different transcriptional profiles, making it a potentially useful drug target [69,70,72]. There are currently no drugs in use that directly target c-Myc [73], so synthetic lethality with TRIB1 may be an avenue worth pursuing, given the apparent druggability of TRIB1 revealed by the structural studies described above.

#### 4.2.3. Regulation of Cell Cycle Progression in Breast Cancer

Bayesian network modelling of differentially expressed genes in breast cancer cells suppressed in the G_1_ phase of the cell cycle has identified TRIB1 as a novel cell cycle regulator in triple-negative breast cancer cells [21]. Predictions from the statistical modelling were confirmed by siRNA knockdown, with TRIB1 knockdown resulting in G_1_ phase enrichment in MDA-MB-231 cells [21]. The knockdown of TRIB1 also increased spontaneous apoptosis across a variety of breast cancer cell lines and sensitised the same cell lines to TRAIL treatment [21]. Regulation of the cell cycle is purported to occur through the regulation of important cell cycle regulators, such as CCND1, which are regulated via the modulation of NF-κB [21]. While TRIB1 cell cycle regulation in humans is CDC25-independent, the cell cycle regulation does suggest conservation of general functionality from *Drosophila*, indicating TRIB1 may play an important role in regulating the cell cycle during development.

#### 4.2.4. Regulation of the Tumour Microenvironment

The tumour microenvironment is fundamental to the development, progression and drug response of tumours and is regulated by a number of factors, including the excretion of cytokines by tumour cells [74,75]. In prostate cancer, the overexpression of TRIB1 promotes cytokine secretion from the tumour cells, which drives the polarisation of M2-like tissue-resident macrophages in the tumour microenvironment [62]. M2-like macrophages are important protumour, anti-inflammatory macrophages that can suppress the local immune response and promote tumour growth [62,74,75]. TRIB1 overexpression promotes the secretion of IL8 and CXCL2, among other cytokines, from prostate cancer cells by inhibiting the NF-κB inhibitor IKB-zeta [62]. The release of these cytokines promotes the M2-like macrophage polarisation, resulting in a protumour environment with low levels of proinflammatory IL12 and high levels of the anti-inflammatory IL10 [62,76].

### 4.3. TRIB1 and Treatment Resistance

TRIB1 overexpression is associated with treatment resistance in both haematological and solid tumour cancer types [21,39,44,45,77]. In PML/RARA-driven APL, TRIB1 overexpression significantly inhibits ATRA-driven differentiation, leading to ATRA resistance [53]. TRIB1 overexpression has also been implicated as central to the development of treatment resistance in a range of solid tumour types [44,45,77]. In triple-negative breast cancer cells, TRIB1 was one of only six genes upregulated in both MDA-MB-231 and HS578T paclitaxel-resistant cells, indicating it is likely essential in the development of paclitaxel resistance [77]. In non-small lung cancer cells, cisplatin treatment resulted in the enrichment of multidrug-resistant cancer stem cells, which overexpress TRIB1 [44]. The knockdown of TRIB1 resulted in the downregulation of the key cancer stem cell promoting transcription factors and the resensitisation of the cancer stem cells to cisplatin [44], confirming the importance of elevated TRIB1 expression in the development of the drug-resistant cancer stem cells. TRIB1 knockdown has also been shown to sensitise MCF7 cells to nutlin-3 treatment [39] and to sensitise various triple-negative breast cancer cell lines, including MDA-MB-231 cells, to TRAIL-induced apoptosis [21], further implicating TRIB1 in drug response in breast cancer. In glioma, TRIB1 overexpression drives the silencing of p53 and promotes resistance to radiotherapy [45]. The association of increased TRIB1 expression with treatment resistance, and decreased TRIB1 with sensitisation to treatment, across cancer types reiterates the importance of TRIB1 in oncogenesis and therapy in a range of contexts. 

## 5. Future Directions 

TRIB1 is emerging as a critical player in both tumour development and treatment resistance across a variety of cancers. The emerging roles of TRIB1 in solid tumours highlight the need for further structural investigation of nondegradative TRIB1 mechanisms of action, which are currently poorly understood. In lieu of directly targeting TRIB1, the pervasive roles of TRIB1 and other Tribbles in oncogenesis and treatment response offer potential as biomarkers for therapeutic choice. In the context of various levels of TRIB1 expression in different solid tumour types [78,79], more systematic studies will be required to establish which subclasses of cancer TRIB1 levels could potentially aid prognosis. In the longer term, identification of key structural differences between degradative and nondegradative mechanisms may allow for the selective drugging of specific TRIB1 functions. Structural understanding of different states of TRIB1 already offers the promise that such compounds could be possible.
